# Disease progression in probable limbic‐predominant age‐related TDP‐43 encephalopathy

**DOI:** 10.1002/alz.71954

**Published:** 2026-11-05

**Authors:** Charlotte Moss, Lei Yu, Konstatinos Arfanakis, Ajay Sood, for Alzheimer's Disease Neuroimaging Initiative

**Affiliations:** ^1^ Rush Alzheimer's Disease Center Rush University Medical Center Chicago Illinois USA; ^2^ Department of Neurological Sciences Rush University Chicago Illinois USA; ^3^ Department of Biomedical Engineering Illinois Institute of Technology Chicago Illinois USA

**Keywords:** Alzheimer's disease, cognitive assessments, dementia, limbic‐predominant age‐related TDP‐43 encephalopathy, mild cognitive impairment

## Abstract

**INTRODUCTION:**

Limbic‐predominant age‐related transactive response DNA‐binding protein of 43 kDa (TDP‐43) encephalopathy (LATE) is confirmed in *post mortem* examination by TDP‐43 pathology. Applying certain criteria, we examined cognitive trajectories in probable LATE and Alzheimer's disease (AD) participants compared with cognitively normal (CN) participants.

**METHODS:**

From the Alzheimer's Disease Neuroimaging Initiative, 108 individuals were classified into AD, probable LATE, or CN groups based on cognitive status, amyloid beta/tau biomarkers, and hippocampal volume. Cognition was assessed prospectively after baseline.

**RESULTS:**

Both probable LATE and AD showed faster decline than CN participants. The magnitude of decline was generally greater in AD than in probable LATE across cognitive domains. Annual rates of change were 0.81 and 2.08 for probable LATE and AD, respectively, on the Alzheimer's Disease Assessment Scale–Cognitive Subscale, 0.24 and 0.36 on the Clinical Dementia Rating Scale–Sum of Boxes, −0.50 and −0.72 on Logical Memory II, and 5.83 and 10.98 on Trail Making Test Part B.

**DISCUSSION:**

These findings support the identification of probable LATE clinically and may inform diagnostic strategies and therapeutic trial design.

## Highlights

- Probable LATE can be identified based on amnestic presentation, HV loss, and amyloid‐negative status.
- Probable LATE primarily impacts episodic memory and executive function.
- Probable LATE shows slower cognitive decline than AD but faster than normal aging.

## 1 BACKGROUND

Limbic‐predominant age‐related transactive response DNA‐binding protein of 43 kDa (TDP‐43) encephalopathy (LATE) is a neurodegenerative disorder characterized by the pathological accumulation of TDP‐43, resulting in significant hippocampal atrophy^1^. This atrophy, notably in the medial temporal lobe, leads to memory loss similar to the amnestic form of Alzheimer's disease (AD). However, in contrast to AD, individuals with pure LATE lack the presence of amyloid beta (Aβ) and tau pathology, although these pathologies can co‐occur, resulting in mixed‐pathology cases confirmed during an autopsy. Clinically, LATE progresses gradually, leading to more pronounced memory deficits relative to other cognitive domains such as language, perception, or executive function^2^, ^3^, ^4^. Episodic memory is notably impaired early in LATE, while immediate memory remains relatively preserved. Other cognitive domains typically become affected only in later stages of the condition^5^, ^6^, ^7^, ^8^. As compared with AD, individuals with LATE are more likely to be less cognitively impaired at their baseline cognitive testing and have overall higher scores on all neuropsychological assessments^3^, ^9^.

Hippocampal atrophy is a significant attribute of LATE contributing to cognitive decline. Pathological studies have demonstrated that TDP‐43 accumulates around the medial temporal lobe leading to neuronal loss. On magnetic resonance imaging (MRI), this manifests as atrophy and volume loss in the hippocampal area^1^, ^10^, ^11^, ^12^. Several clinico‐pathological studies involving longitudinal *ante mortem* MRI and *post mortem* confirmed TDP‐43 deposits in the medial temporal lobe have shown that TDP‐43 is independently associated with smaller hippocampal volumes^13^, ^14^ and faster rates of hippocampal volume (HV) loss^15^.

LATE is relatively less studied as a condition than AD; Neumann et al. first described the presence of TDP‐43 inclusions in the hippocampus in 2006^16^, but the consensus diagnostic criteria for LATE were published in 2019^1^. Definitive diagnosis for LATE requires an autopsy confirmation of TDP‐43 in the medial temporal region leading to neuronal loss, although in development, no biomarkers currently exist to detect TDP‐43 pathology. However, given the easy access to commercially available AD biomarkers such as Aβ testing via positron emission tomography (PET) scan or cerebrospinal fluid (CSF) analysis, clinicians can diagnose LATE in amnestic patients with negative AD biomarkers and MRI findings of hippocampal atrophy. In the absence of histopathological confirmation, Wolk et al. proposed a pragmatic framework to establish more concrete and standardized guidelines for diagnosing LATE^17^. According to Wolk's criteria, the three primary requirements for a probable LATE diagnosis are impaired cognition, reduced HV, and absence of Aβ pathology on CSF or PET scans^17^. While these criteria may not distinguish cases with coexisting LATE and AD pathology, they can reliably differentiate probable LATE from AD, as advances in the detection and use of AD biomarkers have improved the ability to exclude AD as a potential diagnosis^18^. Although the disease progression of histopathologically confirmed LATE has been documented, few studies have evaluated the progression of probable LATE based on Wolk's criteria. Recent work by Groot et al. has begun to operationalize LATE criteria in vivo^19^. Building on this emerging literature, the present study examines probable LATE in an independent US‐based cohort and extends prior work by including comparisons with cognitively normal (CN) individuals in addition to AD.

**RESEARCH IN CONTEXT**

1. **Systematic review**: The authors reviewed the existing literature of LATE, as well as comparisons between LATE and AD. Studies primarily examined clinical aspects of the condition through administration of cognitive assessments, notably in autopsy studies.
2. **Interpretation**: This study is the first in the United States to identify slower cognitive decline in probable LATE compared with individuals with normal cognition using contemporary imaging and clinical measures, extending prior *post mortem* findings. This decline manifests in global cognition, memory, and executive function domains at a slower rate than individuals with AD, but a steeper decline than CN participants. Our findings highlight the importance of establishing probable LATE as a clinical diagnosis by demonstrating its distinct pattern of progression.
3. **Future directions**: Advancing this area of neurodegenerative research requires future development of TDP‐43 biomarkers. Additionally, comparing progression between autopsy‐confirmed LATE and probable LATE criteria using broader samples is crucial for advancing this emerging area.

Gaining a clearer understanding of the clinical course of LATE in the absence of AD pathology could enhance the identification of affected patients and inform both diagnostic approaches and the design of therapeutic trials for this condition.

In this study, we used data from the Alzheimer's Disease Neuroimaging Initiative (ADNI), and we examined disease progression of probable LATE using Wolk et al.’s criteria^17^ and AD, as defined by AD biomarkers, compared with CN participants. We aim to establish the importance of probable LATE as a clinical diagnosis by demonstrating that its pattern of progression in a group of participants is unique to this condition.

## 2 METHODS

### 2.1 Participants

Participants’ data were sourced from the ADNI database (www.loni.ucla.edu/ADNI). With ADNI phase 4 still in progress, participant eligibility and cohort classification were determined using ADNI2 and ADNI3 data only, as the imaging measures required for classification were available only during these phases. After cohort assignment, all available longitudinal cognitive assessments from ADNI1, ADNIGO, ADNI2, and ADNI3 were included in the analyses (*n* = 2431; Figure 1). All participants provided written informed consent. Inclusion and exclusion criteria for ADNI were previously published^20^. Participants were included if they underwent MRI, Aβ PET, and tau PET scans and if these scans met the quality control standards (*n* = 789). A total of 1642 participants were excluded for not meeting these criteria. For study inclusion, we used data closest to each participant's most recent Aβ PET scan. Individuals with other explicit dementia diagnoses, such as vascular dementia, Parkinson's dementia, or substance abuse, were excluded (*n* = 45).

**FIGURE 1:**
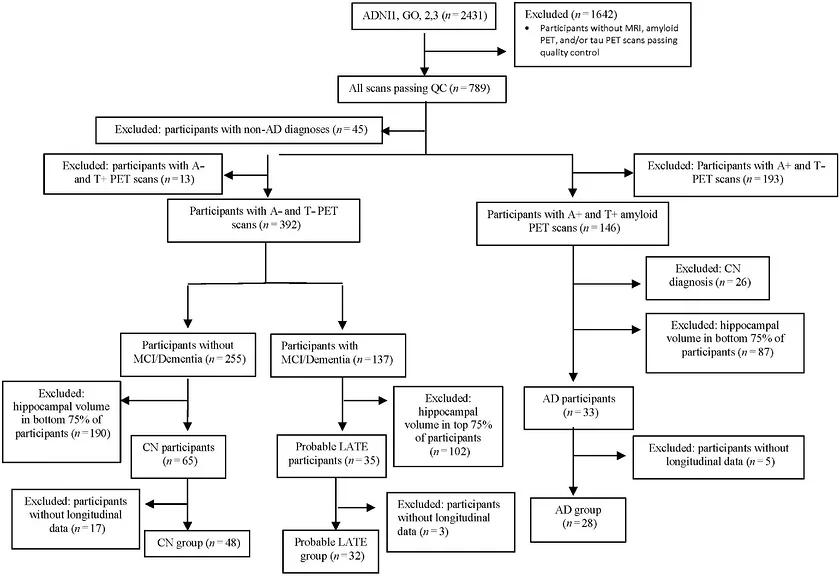
Flow diagram of study participants, including for CN, AD, and probable LATE groups. ADNI, Alzheimer's Disease Neuroimaging Initiative; QC, quality control; A+, amyloid positive; A−, amyloid negative; T+, tau positive; T−, tau negative.

The remainder of the participants (*n* = 744) were divided into two groups based on Aβ and tau status: Aβ and tau‐positive (AD biomarker‐positive) participants (*n* = 146) and AD biomarker‐negative participants (*n* = 392), with participants not meeting either classification excluded from further analysis (*n* = 206). Those with AD‐negative biomarkers were further divided into two groups based on the ADNI diagnosis of CN (*n* = 255) or mild cognitive impairment (MCI)/dementia (*n* = 137), closest to the time of their most recent Aβ PET scan^20^. ADNI criteria for CN, MCI, and dementia are shown in Table 1. Memory impairment in the ADNI was defined as a Clinical Dementia Rating (CDR) memory box score of ≥ 0.5 and a Logical Memory II score of <11 for 16 or more years of education, ≤9 for 8 to 15 years of education, and ≤6 for 0 to 7 years of education. CDR memory box score and Logical Memory II are universally accepted measures of memory assessment and have been used in multiple epidemiological studies and randomized clinical trials.

**TABLE 1:** Criteria for ADNI diagnosis groups. TABLE 1 Criteria for ADNI diagnosis groups.

	CN	MCI	Dementia
MMSE	24 to 30	24 to 30	ADNI1, GO,2: 20 to 26 ADNI3: 20 to 24
CDR	Global = 0	Global = 0.5	Global = 0.5 or 1
	Memory box = 0	Memory box ≥ 0.5	
Logical Memory II	a. ≥9 for 16 or more years of education	a. <11 for 16 or more years of education	ADNI2: No criteria
	b. ≥5 for 8 to 15 years of education	b. ≤9 for 8 to 15 years of education	ADNI3:a. ≤8 for 16 or more years of education
	c. ≥3 for 0 to 7 years of education	c. ≤6 for 0 to 7 years of education	b. ≤4 for 8 to 15 years of education
			c. ≤2 for 0 to 7 years of education
Other		Must express subjective memory complaint	Must express subjective memory complaint
			NINCDS/ADRDA criteria for probable AD

Participants in these three groups (AD‐biomarker positive MCI/dementia, AD‐biomarker negative CN, and AD‐biomarker negative MCI/dementia) were divided into four quartiles based on HVs to rule out other etiologies.

The LATE group included individuals meeting Wolk et al.’s criteria^17^ for probable LATE, with negative Aβ and tau status based on PET scans, significant hippocampal atrophy with HV in the lowest quartile, and an amnestic presentation with ADNI research diagnosis of MCI or dementia at or before the most recent amyloid PET scan. Three participants were excluded for having fewer than two longitudinal assessments, resulting in a final sample of 32 participants.

Participants in the CN group were selected based on negative Aβ and tau PET scans, with the highest quartile of HV to rule out neurodegenerative pathology in the hippocampus and no signs of cognitive impairment (confirmed by a CN research diagnosis in ADNI). Seventeen participants were excluded because they had fewer than two longitudinal cognitive assessments, leaving a final count of 48 participants.

Similarly, the AD group included participants with positive Aβ and tau PET scans having the highest quartile of HV to rule out other mixed co‐pathologies like LATE or hippocampal sclerosis (HS) and a MCI/dementia diagnosis in ADNI. Five participants were excluded for lacking at least two longitudinal assessments, resulting in a final count of 28 participants.

Figures S1 and S2 show the distribution of HVs by group before and after quartiles are applied. As shown in Figure S1, the distribution of HVs (measured at the time of amyloid imaging) demonstrates that AD participants have smaller HVs than CN individuals. Even after restricting the AD group to the highest quartile of HV within the ADNI cohort (Figure S2), these participants still exhibit substantially lower HVs compared with CN individuals (Table 2: normalized HV 0.28 ± 0.02 in AD vs 0.36 ± 0.07 in CN). Thus, our approach did not select individuals with preserved hippocampi but rather those with relatively less severe atrophy within an overall atrophic AD population. By only including participants with the highest quartile of HV among those with positive AD biomarkers, we aimed to maximize the likelihood of excluding co‐pathologies such as LATE or HS and thereby identify individuals most likely to represent relatively pure AD. While we acknowledge that this approach may enrich for a subset of AD patients with relatively milder medial temporal involvement, we believe it provides a pragmatic strategy to reduce pathological heterogeneity in the absence of direct measures of TDP‐43 or other co‐pathologies.

**TABLE 2:** Demographic and imaging characteristics of all participants in study and stratified by group.

	All participants (*n* = 108)	CN (*n* = 48)	LATE (*n* = 32)	AD (*n* = 28)
**Demographics**				
Female, *n* (%)	68 (62.96)	39 (81.25)	10 (31.25)	19 (67.86)
Age (at amyloid PET), years, mean (SD)	73.24 (8.07)	69.11 (7.23)	79 (7.43)	74.89 (6.25)
Age (at baseline), years, mean (SD)	69.83 (7.24)	65.56 (5.41)	73.8 (7.07)	72.78 (6.13)
Education, years, mean (SD)	16.44 (2.37)	16.46 (1.95)	17 (3.01)	15.75 (2.22)
White, *n* (%)	94 (87.04)	40 (83.33)	31 (96.90)	23 (79.31)
Years in study, mean (SD)	4.70 (3.60)	4.55 (2.89)	6.33 (4.63)	3.10 (2.50)
**Imaging**				
Amyloid negative, *n* (%)		48 (100)	32 (100)	0 (0)
Tau negative, *n* (%)		48 (100)	32 (100)	0 (0)
Normalized total hippocampal volume, mean (SD)	0.30 (0.08)	0.36 (0.07)	0.22 (0.030)	0.28 (0.02)
**Cognition**				
Baseline ADAS‐Cog score, mean (SD)	12.10 (7.32)	6.78 (3.90)	14.56 (6.39)	17.37 (7.25)
Baseline CDR‐SB score, mean (SD)	0.84 (1.48)	0.02 (0.10)	1.47 (1.97)	1.54 (1.47)
Baseline MMSE score, mean (SD)	28.36 (1.95)	29.22 (1.01)	28.29 (1.83)	27.18 (2.47)
ADNI diagnosis group at time of amyloid scan, *n* (%)		CN: 48 (100)	CN: 3 (9.38)^*^	CN: 0 (0)
		MCI: 0 (0)	MCI: 24 (75.0)	MCI: 18 (64.29)
		Dementia: 0 (0)	Dementia: 5 (15.63)	Dementia: 10 (35.71)

* Included due to diagnosis of MCI at previous visit.

The majority of ADNI participants remained in the study across more than one phase and have data for up to 16 years. Due to variability in data collection schedules and occasional data exclusions for quality control or other reasons, longitudinal data were available for most, but not all, participants across specific assessments. The average study duration was 3 years for AD with over 20% of the sample with 1 year of follow‐up only, 4.5 years for CN, and 6 years for LATE participants.

### 2.2 Neuropsychological testing

Cognitive and neuropsychological testing encompassing global cognition and specific neuropsychological domains were administered at each participant's in‐person annual visit, beginning with their baseline. For our study, we analyzed global cognition using Clinical Dementia Rating Scale–Sum of Boxes (CDR‐SB), Mini‐Mental State Examination (MMSE), and Alzheimer's Disease Assessment Scale–Cognitive Subscale (ADAS‐Cog). We also analyzed domains of memory with Wechsler Memory Scale Logical Memory II (delayed recall) and Rey Auditory Verbal Learning Test (RAVLT) delayed recall, language using Category Fluency Test, attention/executive function using Trail Making Test Part A (TMT‐A) and Trail Making Test Part B (TMT‐B), and visual perception with Clock Drawing Test. Higher scores indicate better performance for MMSE, Logical Memory II, RAVLT delayed recall, Category Fluency Test, and Clock Drawing Test. Lower scores indicated better performance for ADAS‐Cog, CDR‐SB, TMT‐A, and TMT‐B.

### 2.3 MRI imaging

Each of our participants in ADNI received a MRI scan carried out using a 3.0T scanner during screening and then either annually or biennially. Both high‐resolution T2‐weighted and T1‐weighted MRI sequences were acquired using a standardized protocol^21^. Automated Segmentation of Hippocampal Subfields (ASHS) was applied to the hippocampal T2‐weighted MRI scan by ADNI staff^22^. This method has been proven more effective than typical use of FreeSurfer in providing accurate hippocampal measurements^23^. We considered scans for inclusion with a quality assurance rating of two or higher^22^. Raw left and right HVs were normalized by dividing by the intracranial volume^24^ and multiplying by 100. These normalized volumes were added together to create a total normalized HV.

### 2.4 PET scans

ADNI participants received PET scans at their baseline visit and then every 12 or 24 months to measure Aβ and tau deposition. ADNIGO was the first phase to introduce Aβ scans, and ADNI2 was the first phase to introduce tau scans. Aβ scans used either the florbetapir or florbetaben tracers, and Aβ positivity was determined by previously validated thresholds^25^. Tau scans used the flortaucipir tracer, and tau positivity was determined using a cutoff of standardized uptake value ratio (SUVR) ≥ 1.34 in the temporal meta‐ROI validated by Ioannou et al.^26^ Aβ and tau PET data were preprocessed at a uniform resolution of 6 or 8 mm full width at half maximum using the ADNI pipeline (http://adni.loni.usc.edu/datasamples/pet/; PET uniform resolution information methods). These images were then processed by the ADNI team using their PET analysis pipeline^25^. Data were included in our study if they received a passing or partially passing quality control flag.

### 2.5 Statistical analysis

All statistical analyses were performed using R software (version 4.5.1). Linear mixed‐effects models were used to examine longitudinal change in various cognitive assessment scores across our study's three groups. CN was used as a reference group, and covariates of sex, age, race, and education were added to the model to determine the level of cognition at baseline and annual change of measure. Due to a primarily white sample, we made race binary (White vs non‐White), while age and education remained numerical. The lmerTest package in R was used to run the analysis and calculate *p* values, and statistical significance was set at α = 0.05. A Holm‐Bonferroni correction was performed to control for multiple comparisons. Line plots for slopes derived from the linear mixed‐effects models were generated using the ggplot2 package.

Two sensitivity analyses were performed to evaluate the robustness of the findings. First, hippocampal atrophy was defined using tertiles rather than quartiles to assess the impact of the chosen threshold on group classification. Second, longitudinal analyses of global cognitive outcomes were repeated in a cohort restricted to participants classified as having MCI at their baseline ADNI visit to evaluate whether differences in disease severity influenced the primary findings. The same linear mixed‐effects modeling framework and Holm‐Bonferroni correction procedures used in the primary analyses were applied.

To evaluate the potential contribution of cerebrovascular pathology, a sensitivity analysis was performed incorporating normalized white matter hyperintensity (WMH) volume as a covariate in the mixed‐effects models. WMH volume was obtained from T1‐weighted and fluid‐attenuated inversion recovery MRI scans using a modified Bayesian segmentation approach. WMHs were identified using image intensity, spatial probability maps, and tissue‐class information. Voxels exceeding 3.5 standard deviations above the mean WMH probability were classified as WMHs. WMH volume was normalized by total brain volume and log‐transformed to reduce skewness.

## 3 RESULTS

### 3.1 Participants

A total of 108 participants were included in the analyses, with 32 participants in the LATE group, 48 in the CN group, and 28 in the AD group. Demographic and imaging data closest in time to each participant's most recent Aβ PET scan are shown in Table 2, as well as an ADNI diagnosis group at this time. As expected, HVs were highest in the CN group, followed by AD and LATE groups (Table 2). Additionally, mean baseline scores for global cognition assessments are presented in Table 2. At the time of amyloid PET scan completion, all 48 participants in the CN group were cognitively normal per ADNI neuropsychometric testing criteria. In the probable LATE group, three (9.4%) were CN, 24 (75%) had MCI, and five (15.6%) had dementia. The three probable LATE participants classified as CN at the time of amyloid PET scanning had previously received a diagnosis of MCI in the ADNI study and were included in the sample due to their prior diagnosis, low HV, and the limited number of available participants. In the AD group, 18 participants (64.3%) had MCI, and 10 participants (35.7%) had dementia (Table 2).

### 3.2 Cognitive assessments

Using linear mixed‐effects models controlling for age, sex, education, and race, we examined baseline and annual cognitive decline in global cognition tests across groups. Using the CN group as the reference, both probable LATE and AD groups in our ADNI dataset show lower baseline cognition and faster decline, but AD had the lowest baseline cognition and fastest decline. For ADAS‐Cog, relative to the CN group, participants with AD had higher baseline scores (estimate = 10.15, SE = 1.49, *p* < 0.001) and declined by 2.08 units per year (SE = 0.34, *p* < 0.001). Similarly, compared with the CN group, participants with probable LATE had higher baseline scores (estimate = 7.03, SE = 1.55, *p* < 0.001) and declined by 0.81 units per year (SE = 0.26, *p* < 0.01; Figure 2). A similar pattern was observed for CDR‐SB, with CN as reference: AD participants had a higher baseline score (estimate = 1.31, SE = 0.37, *p* < 0.001) and a steeper decline of 0.36 units/year (SE = 0.12, *p* < 0.05). LATE participants had higher baseline scores (estimate = 1.19, SE = 0.38, *p* < 0.01) and only declined 0.24 units/year (SE = 0.11, *p* < 0.05; Figure 3) compared with the CN group. However, the MMSE did not prove to be very sensitive in measuring disease progression for LATE when compared with CN. The AD group had lower baseline MMSE scores compared with CN participants (estimate = −2.16, SE = 0.46, *p* < 0.001) and declined 1.48 units/year (SE = 0.29, *p* < 0.001). For the LATE group, the MMSE did not reach significance for baseline scores (estimate = −0.52, SE = 0.46, *p* = 0.30) or annual change (estimate = −0.40, SE = 0.26, *p* = 0.26) compared with CN participants (Figure 4). Table 3 presents these results in their entirety. Please refer to Figure S3 (ADAS‐Cog), Figure S4 (CDR‐SB), and Figure S5 (MMSE) for spaghetti plots of all participants and changes in these assessments over time.

**FIGURE 2:**
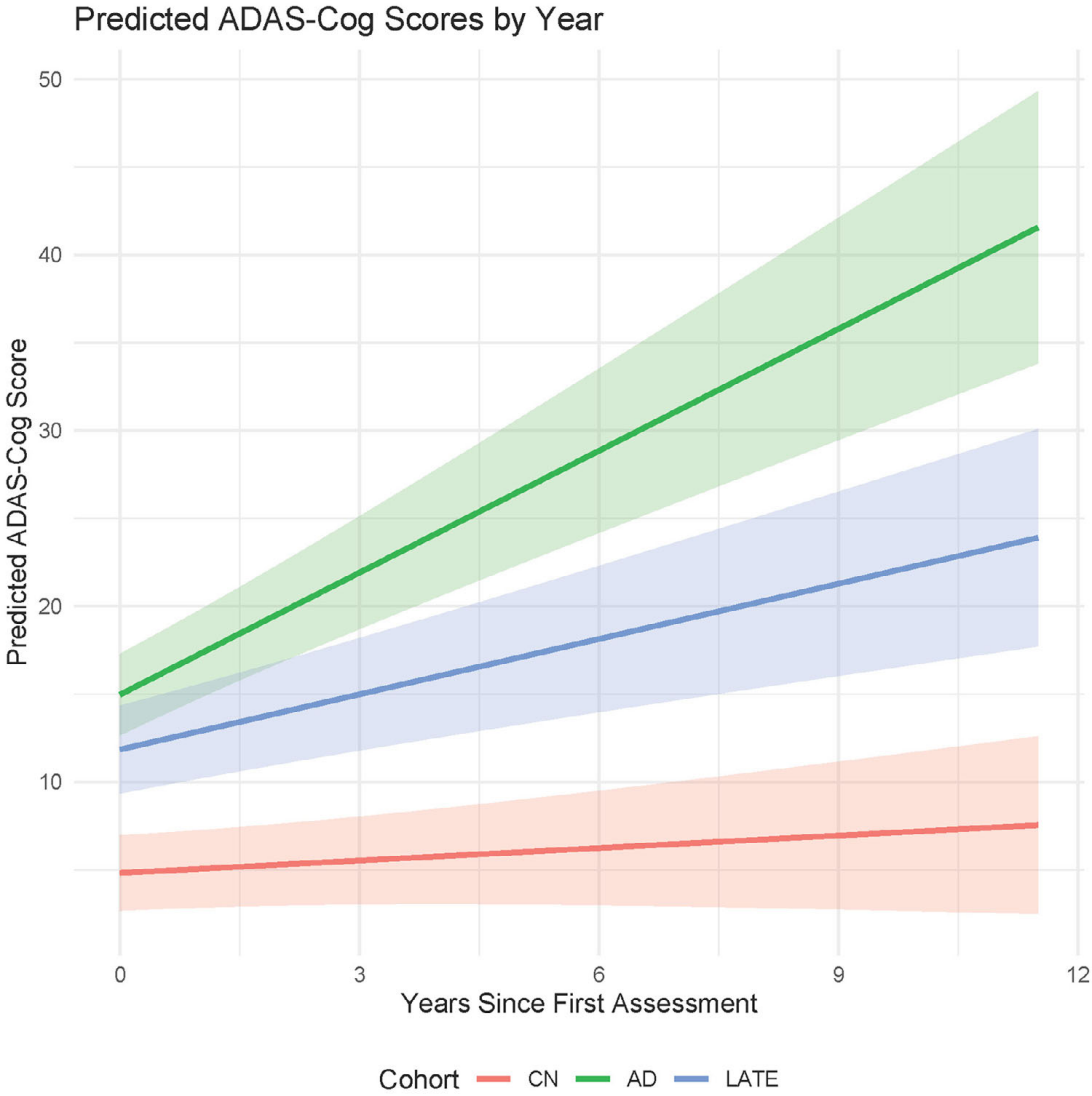
Predicted ADAS–Cog score by year by group (CN: 41 participants, AD: 27 participants, LATE: 31 participants). Shaded area represents 95% confidence interval.

**FIGURE 3:**
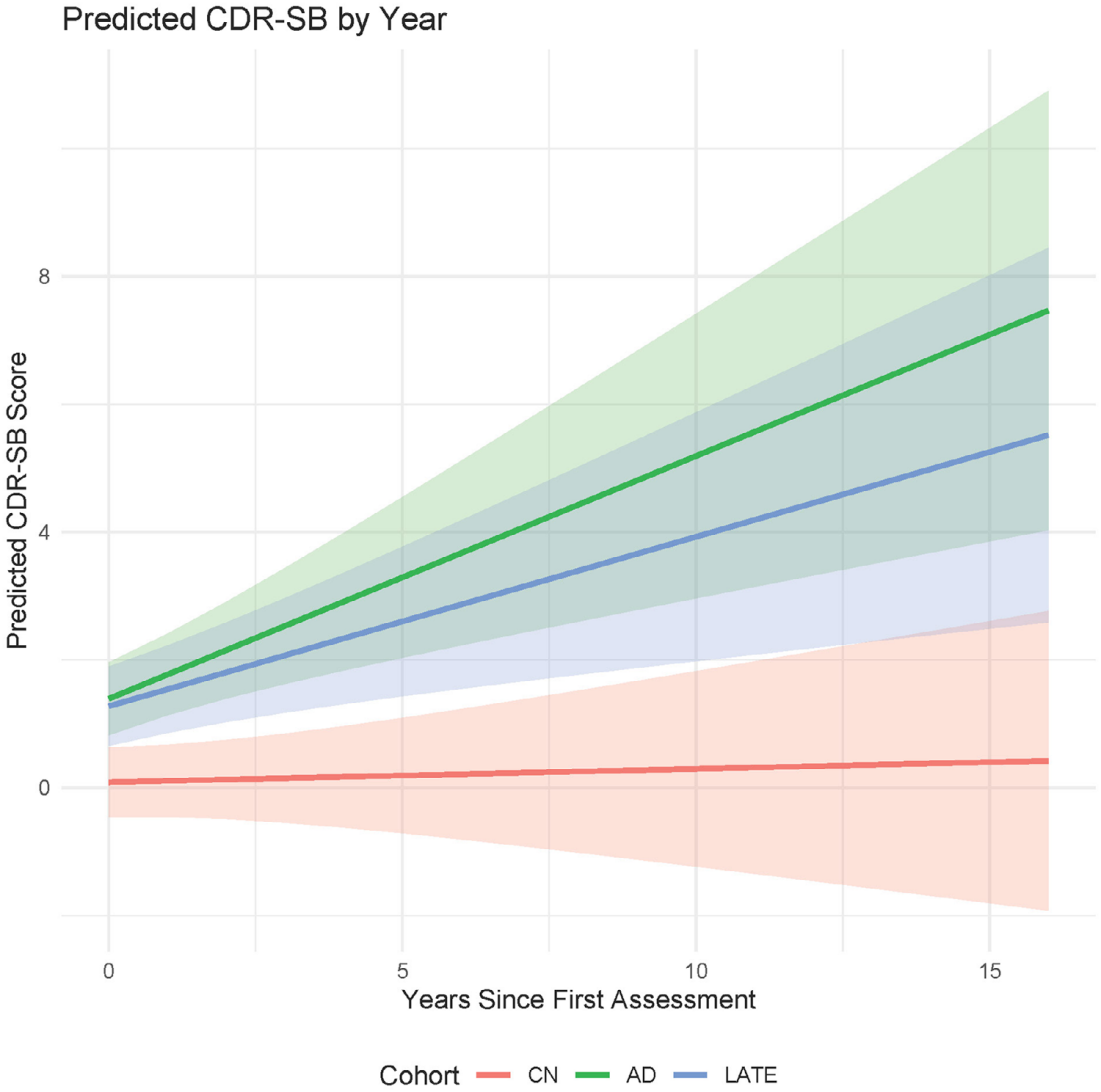
Predicted CDR‐SB score by year by group (CN: 48 participants, AD: 28 participants, LATE: 31 participants). Shaded area represents 95% confidence interval.

**FIGURE 4:**
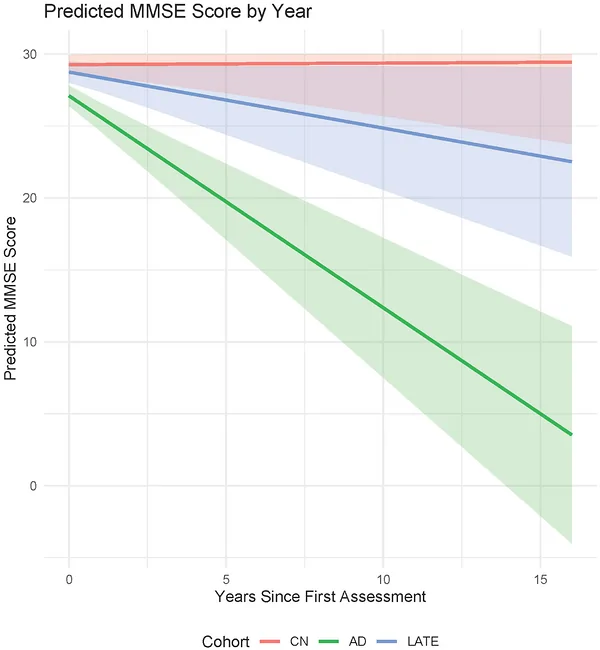
Predicted MMSE score by year by group (CN: 41 participants, AD: 28 participants, LATE: 31 participants). Shaded area represents 95% confidence interval.

**TABLE 3 alz71954-tbl-0003:** Global assessments; CN (*n* = 41), LATE (*n* = 30), and AD (*n* = 27).

Cognitive Assessment		Estimate	Standard Error	Raw p‐value	Holm‐Bonferroni corrected p‐value
ADAS‐Cog	LATE	7.03	1.55	p<0.001	p<0.001
	LATE × year	0.81	0.26	p<0.01	p<0.01
	AD	10.15	1.40	p<0.001	p<0.001
	AD × year	2.08	0.34	p<0.001	p<0.001
CDR‐SB	LATE	1.19	0.38	p<0.01	p<0.01
	LATE × year	0.24	0.11	p<0.05	p<0.05
	AD	1.31	0.28	p<0.001	p<0.001
	AD × year	0.36	0.12	p<0.01	p<0.05
MMSE	LATE	−0.52	0.46	p=0.27	p=0.30
	LATE × year	−0.40	0.26	p=0.13	p=0.26
	AD	−2.16	0.46	p<0.001	p<0.001
	AD × year	−1.48	0.29	p<0.001	p<0.001

*Note*: Rows 3, 5, 7, 9, 11, and 13 depict differences in baseline compared to CN group. Rows 2, 4, 6, 8, 10, and 12 depict rate of decline per year compared to CN group. Linear mixed models adjust for age, sex, education, and race. For ADAS‐Cog, and CDR‐SB, higher scores depict greater cognitive impairment.

Abbreviations: ADAS‐Cog, Alzheimer's Disease Assessment Scale–Cognitive Subscale; CDR‐SB, Clinical Dementia Scale–Sum of Boxes; MMSE, Mini‐Mental State Examination.

While our primary objective was to evaluate disease progression in individuals with LATE and AD relative to CN participants, we also performed a secondary analysis directly comparing the AD and LATE groups. There was no statistically significant difference in baseline ADAS‐Cog scores between the groups (estimate = 3.15, SE = 1.58, *p* = 0.12). However, AD participants exhibited a greater annual decline on ADAS‐Cog than LATE participants (estimate = 1.26, SE = 0.351, *p* < 0.01). In MMSE analyses, AD participants demonstrated lower baseline scores and a steeper rate of decline than LATE participants (both *p* < 0.01). For CDR‐SB, there were no statistically significant differences in either baseline scores or annual rate of decline between the groups.

A sensitivity analysis was conducted using tertiles rather than quartiles to assess whether our results would be affected by slightly broadening the distribution of hippocampal atrophy to assess the robustness of the sample. Relaxing the HV increased the sample size from 108 to 165. The results of this analysis of decline in global cognition scores were similar to those of the original sample. Relative to CN, AD, and probable LATE decline in CDR‐SB and ADAS‐Cog remained significant, and the decline in MMSE remained non‐significant (Table S1).

Next, we assessed baseline and annual cognitive decline across individual tests measuring memory, language, attention/executive function, and visual perception in these groups using linear mixed models that controlled for age, sex, education, and race. Using the CN group as the reference, both LATE and AD showed lower baseline cognition, with AD exhibiting the lowest baseline performance (Table 4).

**TABLE 4 alz71954-tbl-0004:** Domain‐specific assessments; CN (*n* = 41), LATE (*n* = 30), and AD (*n* = 27).

Cognitive assessment		Estimate	Standard error	Raw *p* value	Holm‐Bonferroni corrected *p* value
Logical Memory II	LATE	−4.46	1.00	*p* < 0.001	*p* < 0.001
	LATE × year	−0.50	0.17	*p* < 0.01	*p* < 0.01
	AD	−6.53	0.97	*p* < 0.001	*p* < 0.001
	AD × year	−0.72	0.22	*p* < 0.01	*p* < 0.01
RAVLT delayed	LATE	−5.24	1.67	<0.01	*p* < 0.01
	LATE × year	−0.22	0.14	*p* = 0.14	*p* = 0.29
	AD	−6.56	1.49	*p* < 0.001	*p* < 0.001
	AD × year	−0.27	0.19	*p* = 0.16	*p* = 0.29
Category fluency	LATE	−5.29	2.52	*p* < 0.05	*p* = 0.11
	LATE × year	−0.32	0.16	*p* = 0.06	*p* = 0.11
	AD	−8.12	2.25	*p* < 0.001	*p* < 0.01
	AD × year	0.34	0.22	*p* = 0.14	*p* = 0.14
TMT‐A (s)	LATE	15.50	7.46	*p* < 0.05	*p* = 0.08
	LATE × year	1.80	1.09	*p* = 0.11	*p* = 0.11
	AD	25.32	6.75	*p* < 0.001	*p* < 0.001
	AD × year	5.98	1.23	*p* < 0.001	*p* < 0.001
TMT‐B (s)	LATE	69.31	25.58	*p* < 0.01	*p* < 0.05
	LATE × year	5.83	2.14	*p* < 0.01	*p* < 0.05
	AD	105.75	22.88	*p* < 0.001	*p* < 0.001
	AD × year	10.98	2.71	*p* < 0.001	*p* < 0.001
Clock drawing	LATE	−0.88	0.38	*p* < 0.05	*p* = 0.06
	LATE × year	0.01	0.03	*p* = 0.84	*p* = 0.84
	AD	−1.25	0.34	*p* < 0.001	*p* < 0.001
	AD × year	−0.06	0.04	*p* = 0.14	*p* = 0.28

Linear mixed models adjust for age, sex, education, and race.

Abbreviations: RAVLT, Rey Auditory Verbal Learning Test, TMT‐A, Trail Making Test Part A, TMT‐B, Trail Making Test Part B.

When comparing the annual decline rates in the memory domain, we found that both AD and LATE participants experienced a greater decline in Logical Memory II relative to the CN participants. However, in the RAVLT recall, there were no significant differences in decline between the AD and CN groups (*p* = 0.29) or between the LATE and CN groups (*p* = 0.29). A sensitivity analysis was completed to assess whether percent forgetting, a different output of RAVLT recall, would affect significance in annual decline between groups. This output, calculated as the word recall score at trial 5 minus the delayed recall score, divided by the trial 5 score, did not change the statistical significance of the results: There were no significant differences in decline between the AD and CN groups (*p* = 0.37) or between the LATE and CN groups (*p* = 0.25; Table S2).

For executive function/attention domains, both AD participants and LATE participants showed greater annual decline rates on the performance on TMT‐B compared with CN participants.

For TMT‐A, the AD group demonstrated a steeper annual decline compared with the CN group. However, the decline in the LATE group compared with the CN group was not significant (*p* = 0.11).

The annual rate of decline was not significant for language measured with the Category Fluency Test for AD (*p* = 0.14) compared with CN or for LATE (*p* = 0.11) compared with CN. Perception measured with the Clock Drawing Test showed no statistical difference in rate of change in AD (*p* = 0.28) and LATE (*p* = 0.84) groups as compared with CN. Overall, despite these non‐significant findings, the annual decline in each test was more pronounced in AD participants compared with those in the LATE group (Table 3) compared with the CN group.

To evaluate the potential influence of differences in disease severity at baseline between groups, an additional sensitivity analysis was performed among participants classified as having MCI at baseline (CN = 41, LATE = 28, AD = 20). Baseline differences between the LATE and AD groups compared with CN participants remained significant across global cognitive measures (Table S3). Annual rates of decline on MMSE and ADAS‐Cog remained significantly greater in both disease groups compared with CN participants. The only change in statistical significance was observed for annual change in CDR‐SB in the LATE group, where the Holm‐Bonferroni corrected *p* value increased from 0.03 in the primary analysis to 0.06 in the MCI‐only analysis. Overall, the pattern of findings was similar to that observed in the primary analyses. To evaluate the potential influence of cerebrovascular pathology, an additional sensitivity analysis was performed incorporating normalized WMH burden as a covariate (CN = 41, LATE = 30, AD = 27). The overall pattern of findings remained unchanged (Table S4). Baseline group differences and longitudinal rates of change on ADAS‐Cog and CDR‐SB remained significant after adjustment for WMH burden, suggesting that cerebrovascular disease burden did not materially influence the primary findings.

## 4 DISCUSSION

In this study, we investigated cognitive decline in 108 ADNI participants, including biomarker‐confirmed AD (positive Aβ and tau PET), CN (biomarker negative with limited medial temporal atrophy), and probable LATE (amnestic, Aβ and tau negative, with hippocampal atrophy). At the time of the Aβ PET, HVs were lowest in probable LATE, followed by AD, then CN. Relative to CN, both LATE and AD showed lower baseline cognition and faster cognitive decline in our dataset, with AD exhibiting the lowest baseline performance and the steepest decline, as measured by the ADAS‐Cog and CDR‐SB assessments. The MMSE was sensitive in detecting significant decline only in AD. At baseline, both AD and LATE participants scored lower than the CN participants; however, only AD individuals showed a steeper decline. In a secondary analysis directly comparing AD and probable LATE participants, AD generally demonstrated more rapid cognitive decline; however, differences were not observed across all measures, which may reflect differences in test sensitivity and the relatively small sample size.

The annual decline in the Logical Memory II and TMT‐B assessments was significant for LATE but occurred at a slower rate than in AD relative to CN; however, results from other tests did not reach significance. Overall, these findings indicate a decline in global cognition, memory, and executive function among participants with probable LATE. A sensitivity analysis restricted to participants with MCI at baseline yielded similar findings, with the exception of annual change in CDR‐SB in the LATE group, which no longer met the threshold for statistical significance after correction.

Our data[Table alz71954-tbl-0003], [Table alz71954-tbl-0004] do not show a decline in visuospatial, language, and simple executive function domains over an average observation period of 6 years. However, the small sample size and large standard errors limited statistical power and increased the risk of type II error. Therefore, non‐significant findings in visuospatial, language, and simple executive domains should not be interpreted as evidence of no association.

Building on Groot et al.^19^, we extend in vivo operationalization of LATE criteria by examining an independent US‐based cohort and providing one of the first longitudinal characterizations of probable LATE compared with CN individuals. This supports the validity of this approach for clinicians managing patients with amnestic symptoms, negative AD biomarkers, and significant hippocampal atrophy on MRI scans, findings that align with the criteria for probable LATE.

Neuropathological studies have compared disease progression between LATE neuropathologic change (LATE‐NC) and AD‐neuropathologic change (ADNC), with findings suggesting that LATE‐NC progresses more slowly than ADNC but more rapidly than normal aging. Additionally, individuals with LATE‐NC display slightly more impaired baseline scores in global assessments compared with individuals without neurodegenerative conditions^3^, ^5^, ^27^, ^28^. Our results largely corroborate this pattern and extend it to living biomarker‐defined patients, supporting the validity of disease progression observed in probable LATE.

These findings support probable LATE as a clinically meaningful syndrome distinct from normal aging and AD and suggest that future clinical trials may consider targeting this population.

In this study, the MMSE was not sensitive enough to show significant differences between the LATE and CN groups. Literature indicates that the MMSE has limited effectiveness in distinguishing between MCI and healthy controls^29^, ^30^. Since most of our LATE participants were classified as MCI based on ADNI research standards and showed slower annual decline than the AD group, this may help explain our results. Memory measures showed some discrepancies: The LATE group exhibited steeper decline on Logical Memory II relative to CN, whereas decline on the RAVLT delayed recall was not significant despite lower baseline scores in both LATE and AD groups relative to CN. This may be related to a floor effect in the delayed scores of the RAVLT at baseline, which could hinder participants’ ability to show decline^31^. Sensitivity analyses (RAVLT percent forgetting) did not change results (Table S2). Differences in recall type (word list vs story), small sample size, and higher AD dropout may also have contributed.

The time spent in the study was greater for those with probable LATE than for the AD group. In fact, over 20% of participants with AD stayed in the study for only 1 year. This explains some of the inconsistencies in our results: We could not detect any decline over time in RAVLT recall, Category Fluency, or Clock Drawing in the AD group. Because participants with probable LATE remained in the study longer, the findings, alongside prior evidence^3^, ^9^, ^19^, ^32^, suggest the possibility of slower cognitive decline and greater longevity, though survival bias may partially explain this observation.

Although LATE is commonly associated with advanced age^1^, ^3^, ^33^, ^34^, biomarker‐supported criteria may facilitate identification earlier in the disease course. Clinicians frequently encounter younger patients presenting with amnestic symptoms, hippocampal atrophy on MRI, and negative Aβ status who meet probable LATE criteria. Our findings provide longitudinal data for clinically defined probable LATE and support continued efforts to develop LATE‐specific biomarkers^35^.

A major strength of this study lies in the meticulous effort to rule out other dementia pathologies and mirror the autopsy studies. All participants underwent quality‐assured Aβ and tau PET scans, with Aβ PET being highly reliable for excluding AD in cognitively impaired individuals^17^, ^36^. While not essential for diagnosing probable LATE, a negative tau PET ruled out primary age‐related tauopathy^37^. To further exclude potential pathologies, we selected participants only with the highest quartile of HV for inclusion in both CN and AD groups. For the CN group, this ruled out conditions such as HS, LATE, and AD, whereas for the AD group, it minimized co‐pathologies such as HS and LATE. In the LATE group, only amnestic participants who were Aβ and tau negative and had the lowest HVs were included in the sample. ADNI clinical evaluations excluded other causes of cognitive impairment, such as Parkinson's disease and stroke.

This study has several limitations. Some cognitive assessments were collected during the COVID‐19 pandemic using modified procedures, which may have affected measurement consistency. Additionally, through neurological examinations conducted by ADNI clinicians, we excluded other clinical diagnoses that impair cognition, such as stroke, Parkinson's disease, and Lewy body disease. However, these assessments cannot differentiate conditions like HS, though participants with seizure disorders (a common presentation with HS) were excluded. Given the frequent overlap of HS with LATE‐NC^1^, ^15^, ^32^, it is likely that some LATE participants had hippocampal sclerosis as a co‐pathology. This represents a limitation in defining probable LATE based on Wolk's criteria, and the development of TDP‐43 biomarkers may help further distinguish these two conditions. Because tau PET may miss low levels of tau pathology and co‐pathologies cannot be excluded without histopathologic confirmation^34^, LATE is designated as “probable.” Another limitation is the predominantly White, educated participants in ADNI, making it difficult to generalize the findings to more diverse populations. Furthermore, because ADNI excludes individuals with certain health conditions for safety reasons, the sample may not represent a broader community‐based population.

Additionally, because HV was used to classify participants, between‐group comparisons are not independent and should be interpreted cautiously due to inherent circularity. This approach helped reduce heterogeneity from co‐pathologies (e.g., distinguishing AD from probable LATE) but limits the interpretability of volume differences. It also differs from frameworks that define AD using only amyloid and tau biomarkers, which may affect generalizability. We could not exclude HV from classification without increasing inclusion of mixed or non‐AD cases. Instead of clinical Medial Temporal Atrophy measures^38^ (unavailable in ADNI), we used quartile‐based HVs; sensitivity analyses with broader tertile‐based cognition groupings increased sample size (108 to 165) and yielded consistent results (Table S1). Future work should validate these findings using biomarker‐based classifications independent of HV.

We prioritized PET‐confirmed amyloid and tau biomarkers to maximize pathological specificity as CSF measures (e.g., phosphorylated tau at threonine 181 [p‐tau181]) may reflect upstream processes and introduce heterogeneity^39^, ^40^, ^41^. Although tau positivity is not required for probable LATE^17^, we included tau biomarkers to better exclude individuals with significant tau burden. Future studies using newer fluid biomarkers (e.g., p‐tau217, MTBR‐tau) may help overcome these limitations.

Adjustment for WMH burden did not meaningfully alter the results, suggesting that comorbid cerebrovascular pathology is unlikely to account for the observed findings. Future studies incorporating *α*‐synuclein biomarkers may further improve characterization of co‐pathologies in probable LATE. The smaller sample size may also explain the group‐specific sex differences in participation, as current research has not found strong evidence that LATE differs by sex or ethnoracial background^34^.

This study highlights probable LATE as a clinically relevant entity that is distinct from CN and AD, characterized by a slower‐progressing amnestic syndrome in older adults that can resemble Alzheimer's dementia. Differentiating it from AD is essential, and expanding access to Aβ PET is vital to prevent misdiagnosis due to overlapping clinical symptoms. Progress in this field of neurodegenerative research necessitates future studies with larger, more diverse cohorts to compare disease progression in autopsy‐confirmed LATE cases versus those meeting probable LATE criteria, alongside the development of novel biomarkers to enhance diagnostic accuracy.

## AUTHOR CONTRIBUTIONS

**Charlotte Moss**: researched data, contributed to the discussion, and wrote, reviewed, and edited the manuscript. **Lei Yu**: reviewed the data and edited the manuscript. **Konstatinos Arfanakis**: reviewed the data and contributed to discussion. **Ajay Sood**: researched data, contributed to the discussion, and wrote, reviewed, and edited the manuscript.

## CONFLICT OF INTEREST STATEMENT

CM, LY, KA, and AS have nothing to disclose. ADNI had received grant from NIH (U19 AG024904). Author disclosures are available in the Supporting Information.

## CONSENT STATEMENT

All participants provided written informed consent

## Supporting information




**Supporting Information**: alz71954‐sup‐0001‐SupMat.pdf

**Supporting Information**: alz71954‐sup‐0002‐ICMJE.pdf
