# Supplementary material for: Disease progression in probable limbic‐predominant age‐related TDP‐43 encephalopathy
Source: Alzheimers Dement. 2026 Nov 5;22(11):e71954. doi: 10.1002/alz.71954 (PMC13740345; doi:10.1002/alz.71954)
Supplement: Supplementary file 2 [file ALZ-22-e71954-s002.pdf]

Supplemental Figure 1

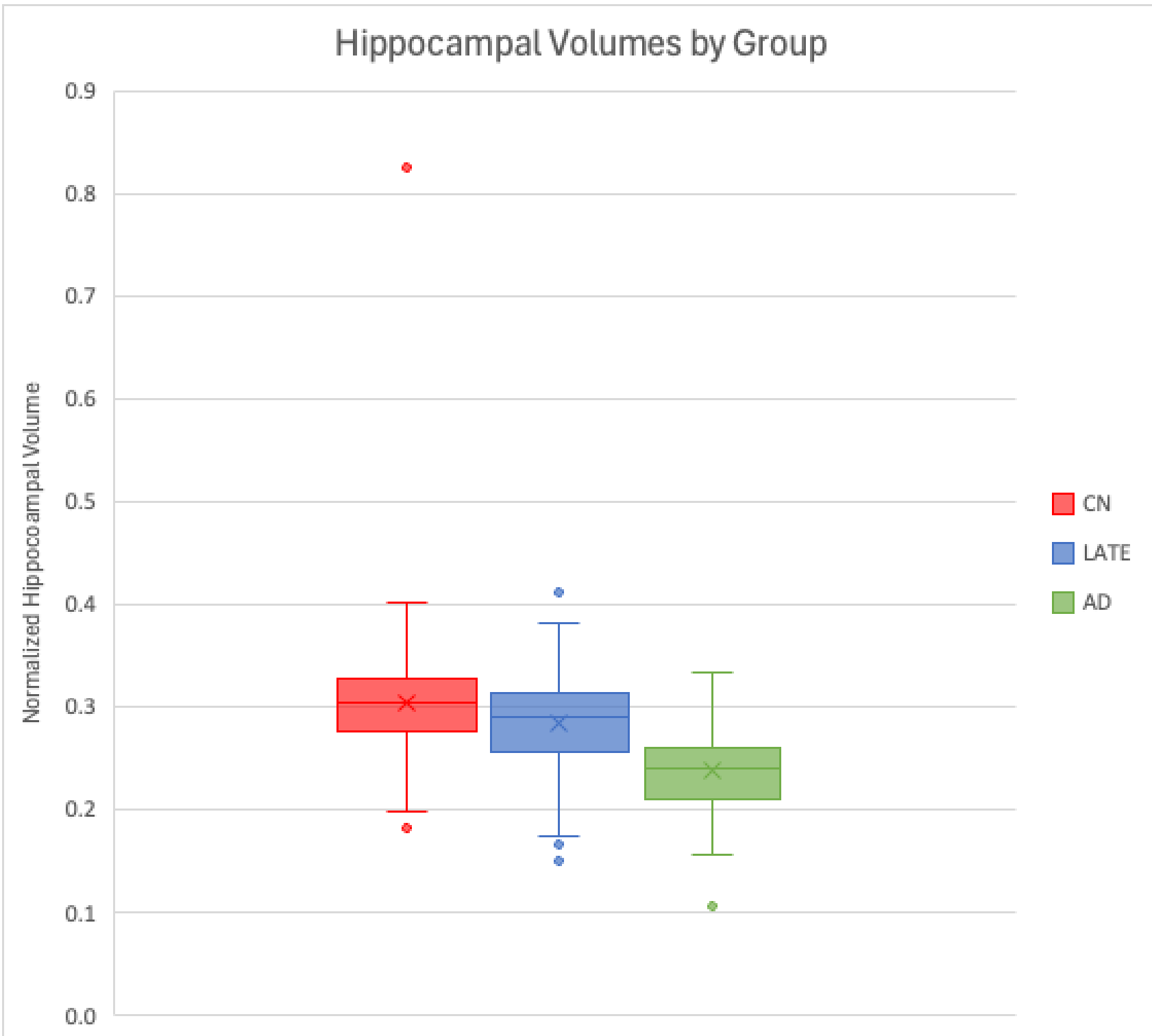

Box plots showing hippocampal volumes of each group before allotment into quartiles

Supplemental Figure 2

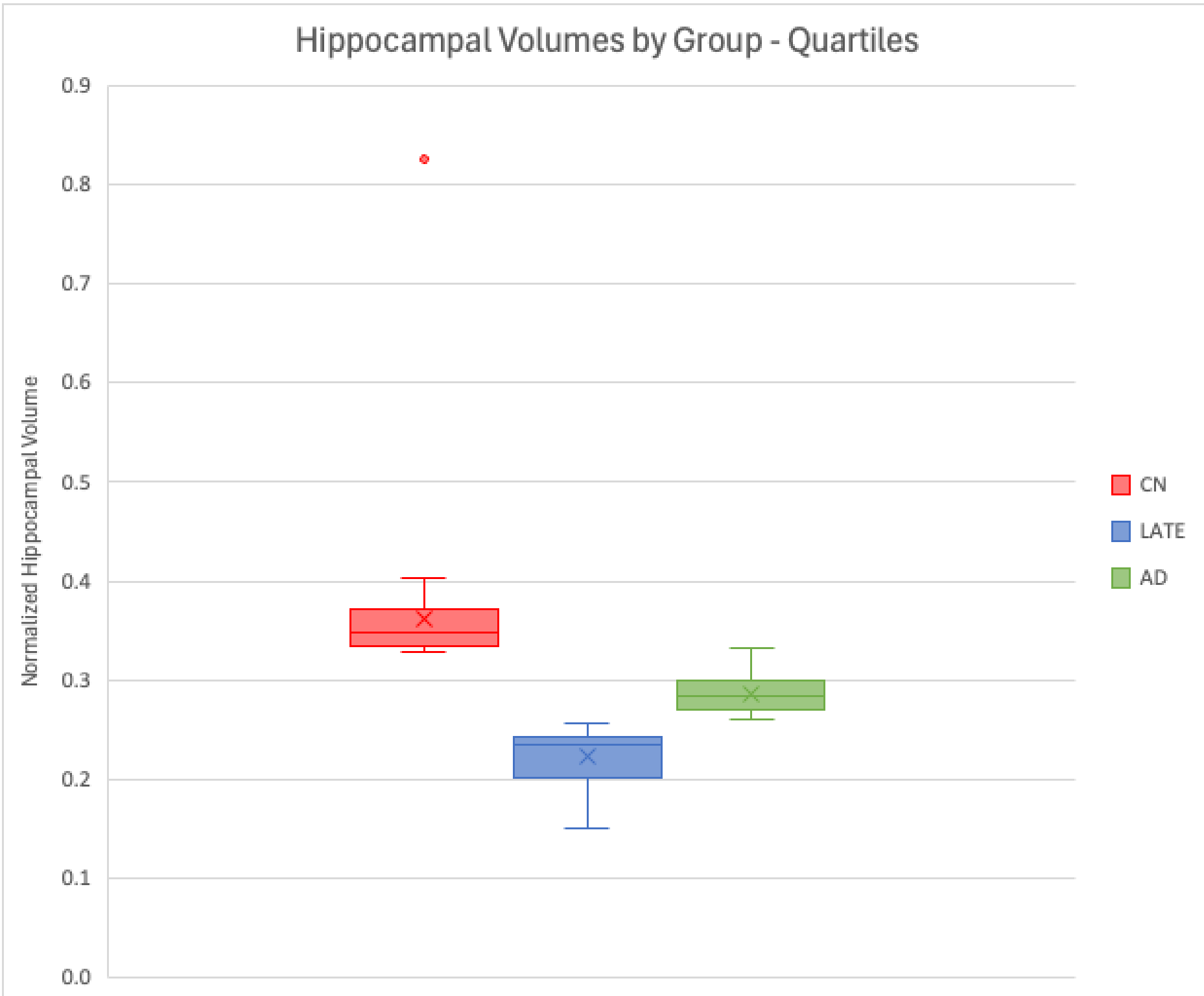

Box plots showing hippocampal volumes of each group after allotment into quartiles

Supplemental Figure 3

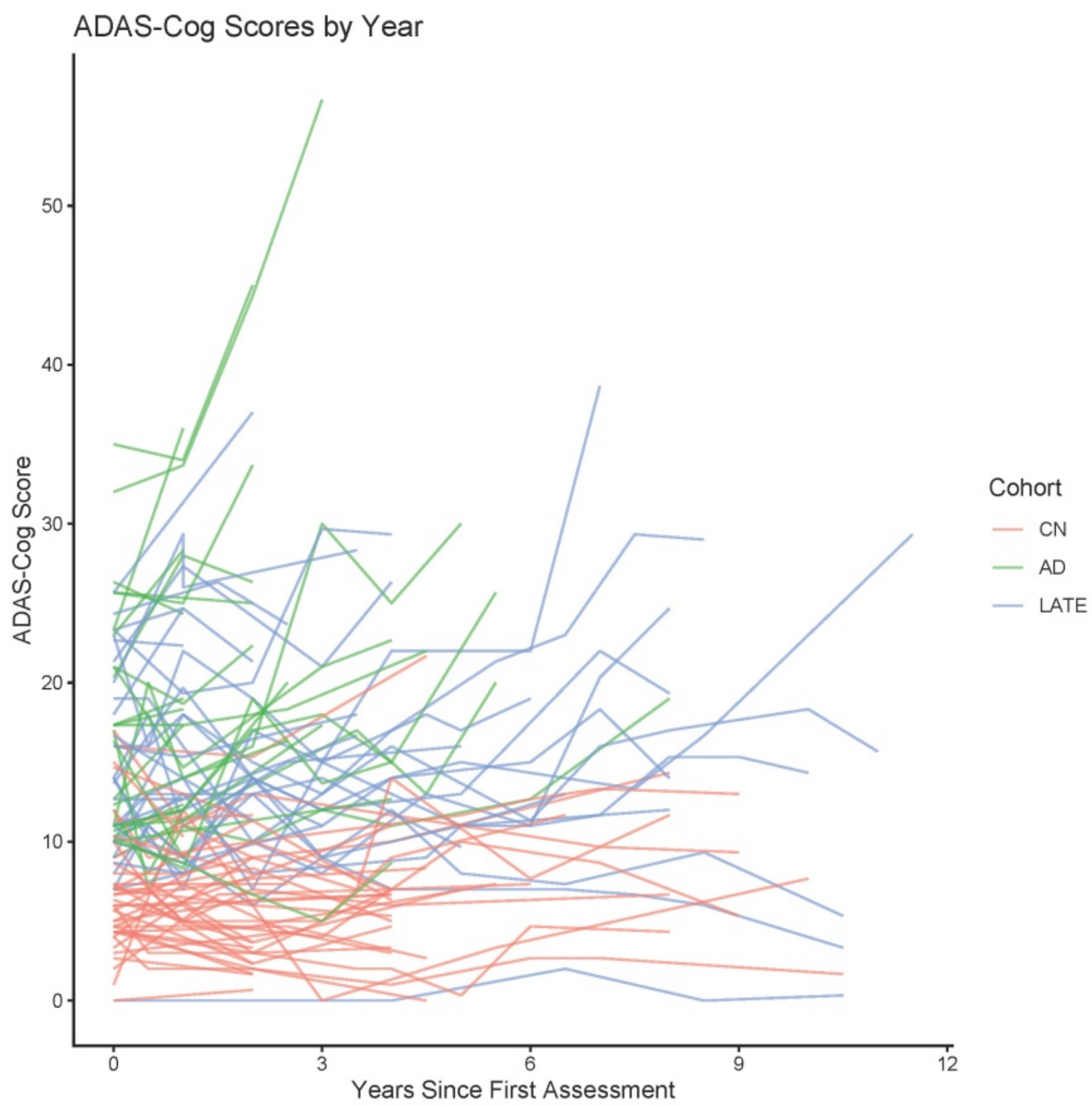

Spaghetti plot showing ADAS-Cog trajectories of individual participants in each group

Supplemental Figure 4

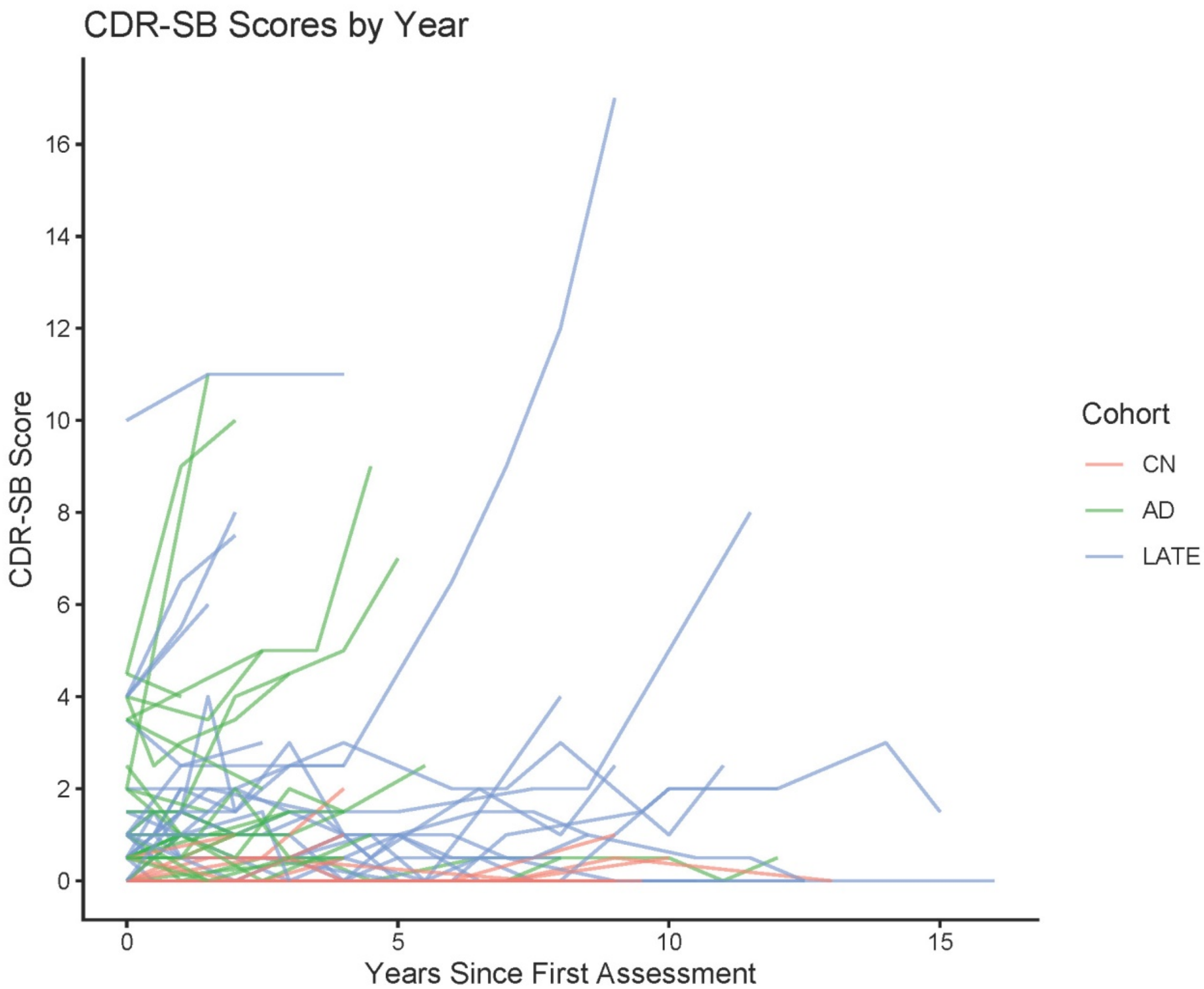

Spaghetti plot showing CDR-sum of the boxes trajectories of individual participants in each group

Supplemental Figure 5

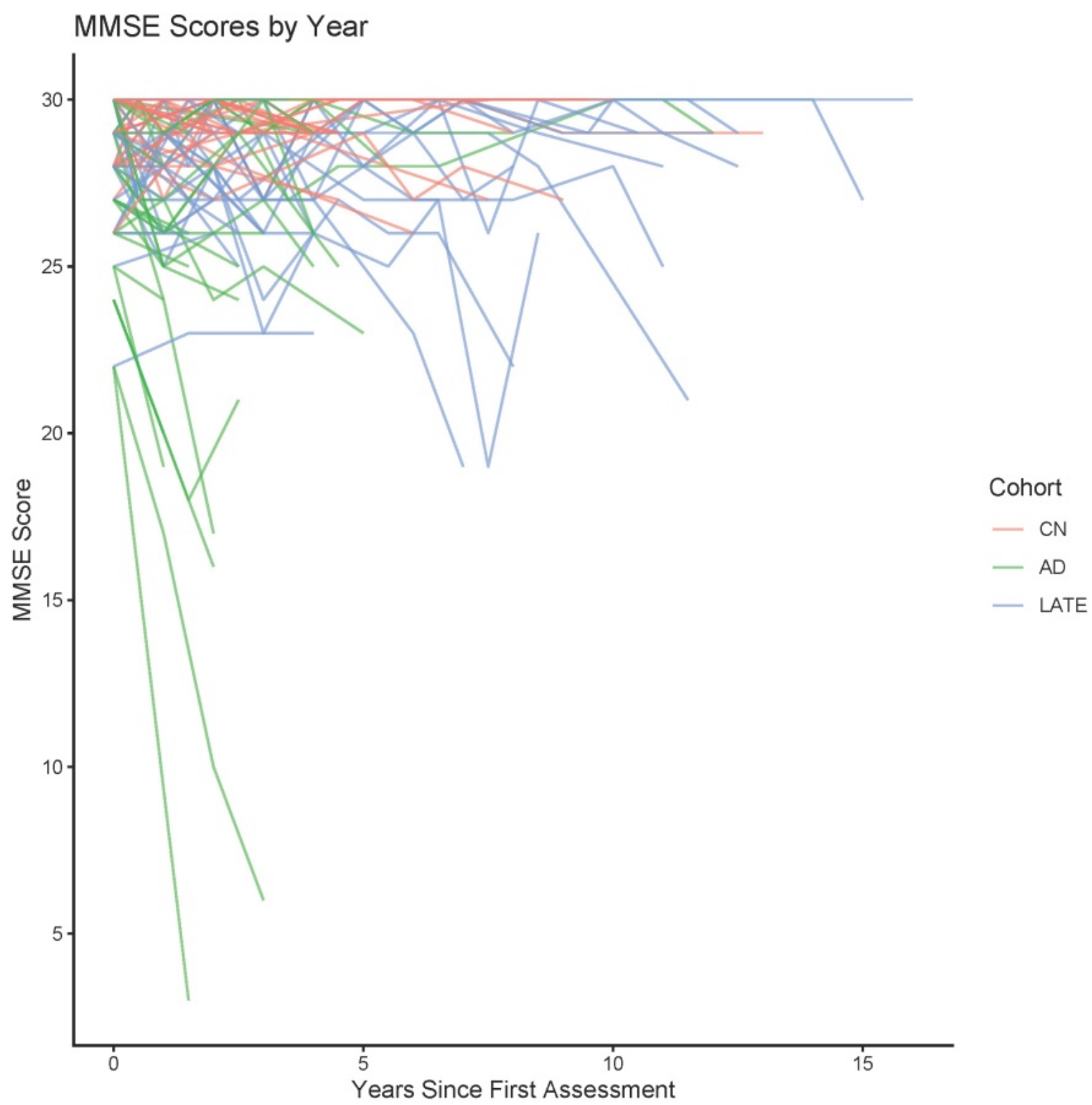

Spaghetti plot showing MMSE trajectories of individual participants in each group

Supplemental Table 1

Cognitive Assessments		Estimate	Standard Error	Raw p-value	Holm-Bonferroni corrected p-value
ADAS-Cog	LATE	6.01	1.16	p<0.001	p<0.001
	LATE × year	0.65	0.20	p<0.01	p<0.01
	AD	9.63	1.07	p<0.001	p<0.001
	AD × year	2.05	0.26	p<0.001	p<0.001
CDR-SB	LATE	1.24	0.29	p<0.001	p<0.001
	LATE × year	0.17	0.08	p<0.05	p<0.05
	AD	1.13	0.27	p<0.001	p<0.001
	AD × year	0.56	0.09	p<0.001	p<0.001
MMSE	LATE	-0.37	0.32	p=0.25	p=0.25
	LATE × year	-0.24	0.14	p=0.09	p=0.18
	AD	-2.02	0.33	p<0.001	p<0.001
	AD × year	-1.07	0.16	p<0.001	p<0.001

Global Assessments Sensitivity Analysis using Tertiles

Rows 3, 5, 7, 9, 11, and 13 depict differences in baseline as compared to CN group
Rows 2, 4, 6, 8, 10, and 12 depict rate of decline per year as compared to CN group
Linear mixed models adjust for age, sex, education, and race.

Abbreviations: ADAS-Cog, Alzheimer's Disease Assessment Scale – Cognitive Subscale, CDR-SB, Clinical Dementia Scale – Sum of Boxes, MMSE, Mini-Mental State Exam

For ADAS-Cog, and CDR-SB, higher scores depict greater cognitive impairment

Supplemental Table 2

	Estimate	Standard Error	p-value
LATE	0.38	0.13	$p<0.01$
LATE × year	0.14	0.02	$p=0.25$
AD	0.45	0.12	$p<0.001$
AD × year	0.16	0.02	$p=0.37$

Rey Auditory Verbal Learning Test (RAVLT) Sensitivity Analysis Using Percent Forgetting

Supplemental Table 3

Cognitive Assessment		Estimate	Standard Error	Raw p-value	Holm-Bonferroni corrected p-value
ADAS-Cog	LATE	6.39	1.43	p<0.001	p<0.001
	LATE × year	0.60	0.26	p<0.05	p<0.05
	AD	8.74	1.46	p<0.001	p<0.001
	AD × year	2.07	0.37	p<0.001	p<0.001
CDR-SB	LATE	0.96	0.25	p<0.001	p<0.01
	LATE × year	0.16	0.08	p=0.06	p=0.06
	AD	0.97	0.26	p<0.001	p<0.01
	AD × year	0.32	0.11	p<0.01	p<0.01
MMSE	LATE	-0.31	0.41	p=0.44	p=0.44
	LATE × year	-0.36	0.17	p<0.05	p=0.08
	AD	-1.66	0.41	p<0.001	p<0.001
	AD × year	-0.83	0.21	p<0.001	p<0.001

Global assessments sensitivity analysis for MCI participants compared to CN (CN=41, LATE = 28, AD=20)

Supplemental Table 4

Cognitive Assessment		Estimate	Standard Error	Raw p-value	Holm-Bonferroni corrected p-value
ADAS-Cog	LATE	7.08	1.57	p<0.001	p<0.001
	LATE × year	0.82	0.26	p<0.01	p<0.01
	AD	10.22	1.51	p<0.001	p<0.001
	AD × year	2.08	0.34	p<0.001	p<0.001
CDR-SB	LATE	1.26	0.41	p<0.01	p<0.01
	LATE × year	0.25	0.11	p<0.05	p<0.05
	AD	1.26	0.39	p<0.01	p<0.01
	AD × year	0.38	0.13	p<0.01	p<0.01
MMSE	LATE	-0.51	0.46	p=0.28	p=0.28
	LATE × year	-0.40	0.26	p=0.13	p=0.26
	AD	-2.15	0.46	p<0.001	p<0.001
	AD × year	-1.49	0.29	p<0.001	p<0.001

Global Assessments Sensitivity Analysis adding White Matter Burden as a Covariate; CN (n=41), LATE (n=30), and AD (n=27)

Rows 3, 5, 7, 9, 11, and 13 depict differences in baseline as compared to CN group
Rows 2, 4, 6, 8, 10, and 12 depict rate of decline per year as compared to CN group
Linear mixed models adjust for age, sex, education, race and WMH burden.

Abbreviations: ADAS-Cog, Alzheimer's Disease Assessment Scale – Cognitive Subscale, CDR-SB, Clinical Dementia Scale – Sum of Boxes, MMSE, Mini-Mental State Exam

For ADAS-Cog, and CDR-SB, higher scores depict greater cognitive impairment
